# Person-Generated Health Data in Simulated Rehabilitation Using Kinect for Stroke: Literature Review

**DOI:** 10.2196/rehab.9123

**Published:** 2018-05-08

**Authors:** Gerardo Luis Dimaguila, Kathleen Gray, Mark Merolli

**Affiliations:** ^1^ Health and Biomedical Informatics Centre University of Melbourne Melbourne Australia; ^2^ Department of Health and Medical Sciences School of Health Sciences Swinburne University of Technology Melbourne Australia

**Keywords:** health care information systems, Kinect, patient-generated health data, person-generated health data, review, simulated rehabilitation, stroke, stroke rehabilitation, video games, virtual rehabilitation

## Abstract

**Background:**

Person- or patient-generated health data (PGHD) are health, wellness, and clinical data that people generate, record, and analyze for themselves. There is potential for PGHD to improve the efficiency and effectiveness of simulated rehabilitation technologies for stroke. Simulated rehabilitation is a type of telerehabilitation that uses computer technologies and interfaces to allow the real-time simulation of rehabilitation activities or a rehabilitation environment. A leading technology for simulated rehabilitation is Microsoft’s Kinect, a video-based technology that uses infrared to track a user’s body movements.

**Objective:**

This review attempts to understand to what extent Kinect-based stroke rehabilitation systems (K-SRS) have used PGHD and to what benefit.

**Methods:**

The review is conducted in two parts. In part 1, aspects of relevance for PGHD were searched for in existing systematic reviews on K-SRS. The following databases were searched: IEEE Xplore, Association of Computing Machinery Digital Library, PubMed, Biomed Central, Cochrane Library, and Campbell Collaboration. In part 2, original research papers that presented or used K-SRS were reviewed in terms of (1) types of PGHD, (2) patient access to PGHD, (3) PGHD use, and (4) effects of PGHD use. The search was conducted in the same databases as part 1 except Cochrane and Campbell Collaboration. Reference lists on K-SRS of the reviews found in part 1 were also included in the search for part 2. There was no date restriction. The search was closed in June 2017. The quality of the papers was not assessed, as it was not deemed critical to understanding PGHD access and use in studies that used K-SRS.

**Results:**

In part 1, 192 papers were identified, and after assessment only 3 papers were included. Part 1 showed that previous reviews focused on technical effectiveness of K-SRS with some attention on clinical effectiveness. None of those reviews reported on home-based implementation or PGHD use. In part 2, 163 papers were identified and after assessment, 41 papers were included. Part 2 showed that there is a gap in understanding how PGHD use may affect patients using K-SRS and a lack of patient participation in the design of such systems.

**Conclusions:**

This paper calls specifically for further studies of K-SRS—and for studies of technologies that allow patients to generate their own health data in general—to pay more attention to how patients’ own use of their data may influence their care processes and outcomes. Future studies that trial the effectiveness of K-SRS outside the clinic should also explore how patients and carers use PGHD in home rehabilitation programs.

## Introduction

### Understanding the Effects of Person-Generated Health Data

Person- or patient-generated health data (PGHD) are health, wellness, and clinical data that people generate, record, and analyze for themselves [1]. Examples of technologies that support PGHD include online health journals, activity-tracking devices or mobile apps, networked health data-gathering devices such as weighing scales, and simulated rehabilitation technologies. However, PGHD integration into clinical use is hampered by the lack of theoretical foundation, strategies, and data models [1]. The availability of PGHD technologies has been increasing, and so has their adoption. However, implementation and evaluation research has not kept up.

PGHD’s effects on the health of the individual have yet to be demonstrated or defined. It is known that when patients understand their illness, they may become active problem solvers and improve their health behavior; for example, it has been suggested that people will stop smoking when they personally see the connection between activity and illness [2]. Moreover, patients’ use of PGHD has been suggested to improve health management coordination between them and their health care providers and treatment teams, assist patients in self-managing their care, engage patients, and increase the social support they receive and their sense of social connectedness [3-8].

In particular, PGHD may make home-based health care more efficient and effective. If not only clinicians but also patients are able to access health data generated from the use of home-based health care technologies, this may improve patients’ engagement in their own care and optimize their use of clinical supervision, thus contributing to more effective outcomes across the health system overall [7,9,10].

PGHD may be especially relevant and accessible to patients who use a particular form of home-based health care, simulated rehabilitation systems. Simulated rehabilitation is a type of telerehabilitation that uses computer technologies and interfaces to allow the real-time simulation of rehabilitation activities or a rehabilitation environment [11]. Users interact with the simulation through multiple sensory channels [12-14].

### Person-Generated Health Data Use Case: Simulated Rehabilitation After Stroke

One important PGHD use case may be in home-based poststroke rehabilitation that uses body-tracking simulated rehabilitation technologies [15]. Stroke is an important application area for rehabilitation systems because of the burden and complexity of the care required. It is a leading cause of death and disability across the globe and accounts for 46.6 million disability-adjusted life years [16,17]. Stroke patient motor function recovery is a long and complicated process, requiring patients to undergo extensive rehabilitation therapy that involves frequent, regular movement exercises matched to their impairments [18,19]. Regular rehabilitation exercises, especially in the first few weeks poststroke, are essential in helping patients recover and reduce long-term impact on their quality of life. However, clinical rehabilitation can be costly and may not be readily available for some patients [20].

More practical and convenient rehabilitation options for patients are needed. Access to an effective home-based rehabilitation program is important in a patient’s journey to recovery. Moreover, patients recovering after a stroke may prefer home-based rehabilitation rather than traveling to a clinic [20]. However, patient compliance with home-based exercise programs may be weak, in part due to the perceived monotony of exercises as well as lack of guidance in completing them [21-23]. The small number of successful trials reporting home-based exercises for stroke are also personnel intensive [20], indicating that therapists’ close involvement remains necessary.

The potential benefit of simulated rehabilitation systems poststroke has been documented in select systematic reviews [12-14,24]. This form of rehabilitation can provide simulation of activities of daily living [24]. At the same time, it can allow the treating therapist a semicontrolled, consistent format for observing and documenting patient performance and progress [24] and for assessing any performance changes [13]. There is potential to decrease rehabilitation costs while increasing accessibility to rehabilitation exercises for patients in areas where there is a dearth of rehabilitation services [21-23]. Since these systems are interactive, many of them gamified, they add enjoyability to exercises, help motivate patients, and encourage adherence to the rehabilitation tasks [24]. As such, they are seen as optimizing the benefits of conventional therapy [12].

Implementing PGHD technologies might further optimize these systems. Simulated setups employ various hardware and software technologies including a range of off-the-shelf technologies [25] to set tasks (which in rehabilitation are often a form of physical exercise), facilitate the accomplishing of tasks, and—crucially for the relevance of PGHD—record the user’s performance [26,27]. Using PGHD tools, performance data could be made accessible to the patient at home and, in Internet-connected settings, could be shared online (in rehabilitation, typically with the therapist) [27].

### Use Case System of Choice: Microsoft Kinect

Microsoft’s Kinect is a video-based technology that uses infrared to track a user’s body movements. It has been suggested as a leading technology for simulated rehabilitation [26] for several reasons: it has good movement range and demands, which helps in rehabilitation; it has been shown to be reliable and accurate; and it demonstrates consistent performance in tracking user movements [28-30]. Also, it is a relatively affordable product that is available to consumers for home entertainment. These factors have led to its adoption for patient therapy in cerebral palsy [31], assessment of foot posture [32], and cardiovascular diseases [33].

Kinect has been used extensively in simulated rehabilitation systems for stroke. Commercial examples used by physical therapists with stroke patients include Limbs Alive and Jintronix. However, little is known about how effectively such systems may facilitate not only clinical data use by therapists but also PGHD use by patients themselves. There is no clear body of evidence about the impact the patient’s experience using PGHD could have on their overall experience of rehabilitation in such systems.

### Objectives of This Review

There is potential to realize greater engagement, efficiency, and effectiveness benefits of Kinect-based stroke rehabilitation systems (K-SRS) deployed in patient’s homes under clinical supervision by allowing each patient to access their own PGHD from the system. Hence, the objective of this review is to answer the questions: To what extent do K-SRS enable PGHD? And to what effect?

## Methods

The literature review was conducted following the guidelines of the Preferred Reporting Items for Systematic Reviews and Meta-Analyses (PRISMA) statement [34], as appropriate to our objectives.

The review is structured in 2 parts:

1. Analysis of existing systematic reviews of K-SRS [25,27,29]. This reveals which aspects of relevance for PGHD have been prioritized by previous studies.
2. Systematic review of the use of PGHD in existing K-SRS.

### Part 1: Analysis of Systematic Reviews

**Figure 1:**
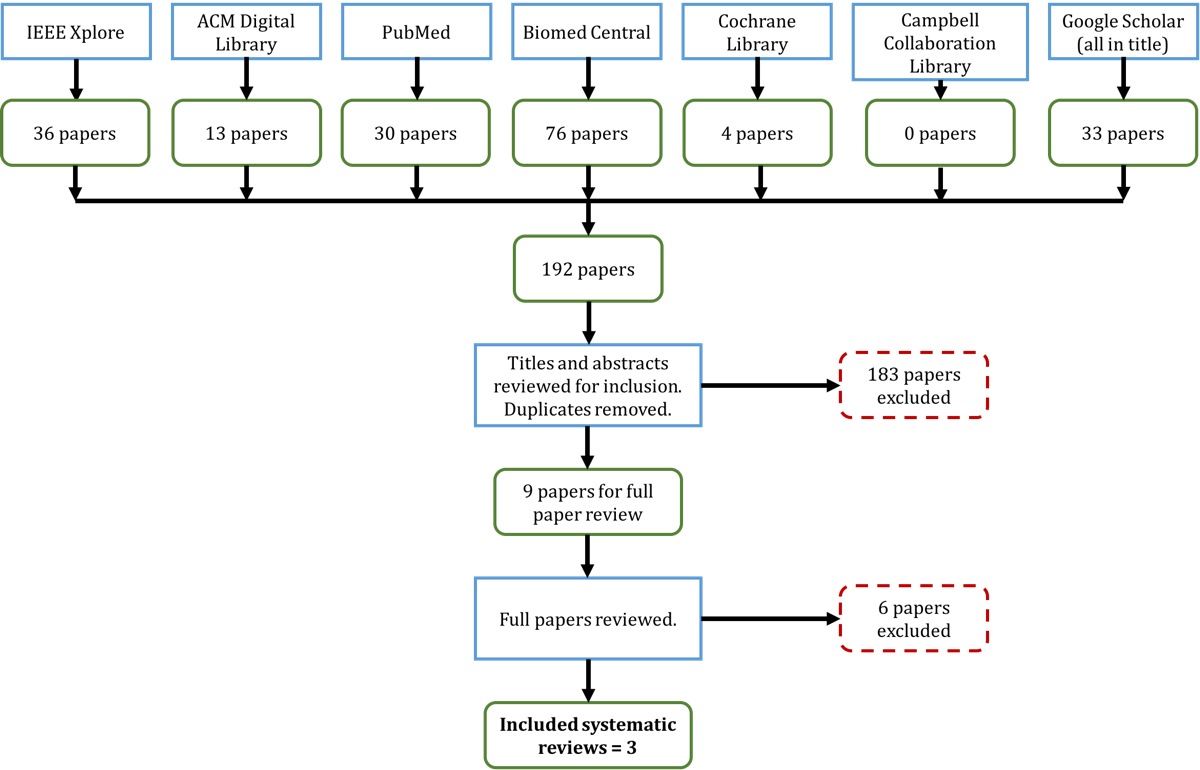
Search process for part 1.

An exhaustive search strategy (Multimedia Appendix 1) resulted in 3 systematic reviews. Figure 1 illustrates this search process. The inclusion criteria include articles written in English, systematic or literature reviews, reviews of systems that used Kinect, and systems for stroke rehabilitation. The exclusion criteria include reviews for nonstroke rehabilitation purposes (eg, assess Kinect’s gesture recognition) or broad scoping reviews that primarily take inventory of a suite of technology-based rehabilitation systems. The content of each systematic review was analyzed based on (1) method for analysis vis-à-vis objectives; (2) focus on use of patient-generated health data, including feedback given to users or patients; (3) the extent to which the systems included in the review are usable at home, including the challenges and recommendations for implementing at home; and (4) the effectiveness of the systems in the review based on patient outcomes as well as technological limitations that may affect those outcomes.

### Part 2: Review of Person-Generated Health Data Use in Kinect-Based Stroke Rehabilitation Systems

**Figure 2:**
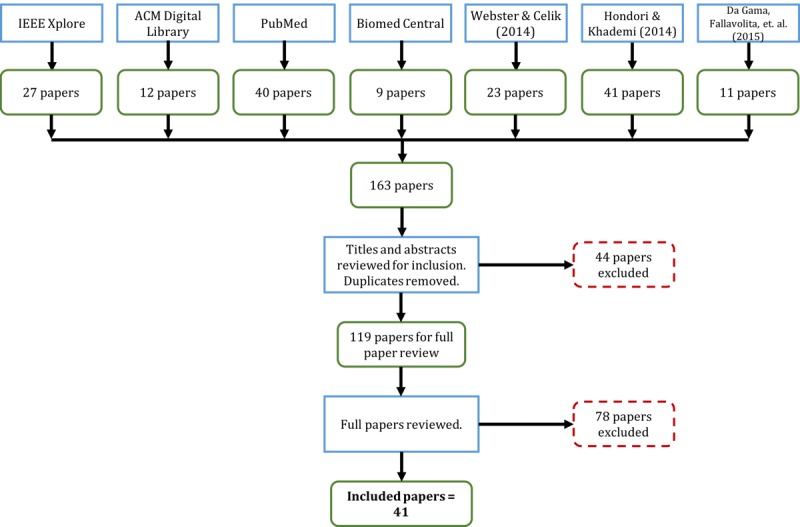
Search process for part 2.

An exhaustive search strategy (Multimedia Appendix 2) resulted in 41 original research reports for review. Figure 2 illustrates this search process. The inclusion criteria include those full papers written in English that present rehabilitation systems for stroke using Kinect. Exclusion criteria include white papers or systematic or literature reviews, primary purpose of study not being rehabilitation or primary disease case not being stroke, or not using Kinect in any way. Based on our objectives, content of the included papers was primarily analyzed using the following questions: (1) What types of data did patients generate? (2) Did they have access to their PGHD, and if yes in what form? (3) How were these data used by patients, clinicians, developers, and researchers? (4) What effects were observed from PGHD use?

## Results

Part 1 highlights gaps in information collected in previous systematic reviews of K-SRS, particularly the use of PGHD and home use of K-SRS. Moreover, it shows that previous reviews mainly provided technical descriptions of K-SRS, while suggesting that more studies are needed to ascertain their clinical effectiveness. Part 2 of this review addresses the PGHD use gap.

### Part 1: Analysis of Systematic Reviews of Kinect-Based Stroke Rehabilitation Systems

The objectives, methods, and structure of each systematic review are detailed in Table A (Multimedia Appendix 3). A summary of these systematic reviews vis-à-vis the themes of interest can be found in Table B (Multimedia Appendix 4).

#### Review of Person-Generated Health Data Use

None of the 3 systematic reviews examined the literature on use or management of patient-generated data, although system feedback methods were briefly described. Webster et al [26], however, noted the need for future research to look into the data-gathering potential of Kinect and provide proper feedback to patients, especially when they fail to accomplish a task. Hondori et al [25] focused on describing the technical and technological aspects and features of Kinect and other body-tracking technologies. Da Gama et al [27] were more comprehensive in their analysis and presentation of papers.

#### Review of Home Use

None of the reviews included a home usability criterion. However, Hondori et al [25] noted the need to assess Kinect’s safety and efficacy when implemented at home. Moreover, Da Gama et al [27] briefly noted some challenges that can be encountered in a home implementation, such as space and lighting conditions. These authors recommended future studies into the effects and benefits of a home-based implementation. How patients might interact with their data outside of clinical settings is a significant gap in our understanding, particularly because K-SRS are touted as beneficial and advantageous for home use [35].

#### Review of Effectiveness

These 3 reviews confirmed the accuracy and reliability of Kinect when used for poststroke rehabilitation, particularly for providing and tracking movement exercises. They also highlighted Kinect’s weaknesses, including occlusion and inability to track fine motor movement such as those including fingers, and suggested that Kinect should be focused only on the whole hand or be used with other technologies such as sensors. Webster et al [26] noted that Kinect may not be suitable for patients with extremely severe impairments because they are only capable of performing minute movements.

All reviews noted that more work is needed to verify the clinical effectiveness of K-SRS and describe their possible benefits for patients. Webster et al [26] discussed the potential physical and mental benefits of K-SRS (ie, faster and better supported rehabilitation and increased enjoyability of exercises and motivation due to the highly interactive interfaces). Kinect-based systems can also extend guidance and correction of patient movements. Moreover, exercises can be tailored to the needs of patients. Hondori et al [25] found that patients preferred Kinect over other off-the-shelf, consumer body-tracking devices, Kinect-based systems can assist in improving balance, and they have the potential to improve functional ability of patients. Da Gama et al [27] echoed the findings of Webster et al [26] that enjoyability increased motivation.

### Part 2: Person-Generated Health Data Use in Kinect-Based Stroke Rehabilitation Systems

Part 1 highlighted gaps in information collected in previous systematic reviews of K-SRS, particularly the use of PGHD and home use of K-SRS. Moreover, it showed that previous reviews mainly provided technical descriptions of K-SRS, while suggesting that more studies are needed to ascertain their clinical effectiveness. Part 2 of this review addresses the PGHD use gap.

#### Article Types

To assist future studies in assessing the clinical effectiveness of K-SRS, papers are categorized as either clinical- or technical-focused. Clinical-focused papers prioritize the clinical effectiveness, feasibility, or safety of K-SRS. They include cohort studies (n=2), case reports (n=2), and randomized controlled trials (n=5). Technically oriented papers prioritize the design, development, and evaluation of the systems. They include a survey (n=1), proofs of concept (n=5), development of an app (n=17) or platform (n=6), and assessment of reliability and precision (n=3). The list of papers categorized according to their clinical or technical type is in Multimedia Appendix 5 (Tables C and D).

#### Participants

##### Health Status

Nearly half of the papers (17/41, 42%) recruited only stroke patients [22,35-50], 15% (6/41) recruited only healthy participants [51-56], and 17% (7/41) recruited both patients and healthy participants [23,30,57-61]. One paper recruited participants for a requirements-gathering phase but no details were provided regarding their health status and demographics [62], and 20% (8/41) of papers did not recruit any participants [63-70]. Both study protocols will be recruiting patients [71,72].

##### Demographics

While 33% (11/33) of papers with subjects did not report ages [45-47,52-54,56,59,60,62,70], there was considerable variation in the ages of both patients and subjects in those that did. For papers that recruited patients, the combined mean age was 59.2 (SD 19.6) years. For papers that recruited healthy subjects, combined standard deviation (39.9 years) was greater than the combined mean (37.3 years), indicative of how spread out the age ranges were. While 38% (9/24) of papers with patients did not record gender [23,44-47,57,59,60,70], the majority of patients in those that did were male and 2 had an equal distribution [39,48]. Meanwhile, 54% (7/13) of papers with healthy subjects did not record gender [52,53,55-57,59,60]; the majority of patients in those that did were male. None had more females, and only 1 had an equal distribution [54].

##### Stroke Details

Only 12% (5/41) of papers recorded the stroke types of patients [38,40-42,49], and of those all patients had infarct or ischemic strokes except for one, which had 10 ischemic and 5 hemorrhagic patients [49]. More than a quarter (11/41, 27%) of papers recorded hemiparetic side of patients [23,36-42,48-50], and the majority of recorded hemiparesis was the right side (7/11, 64%). Only 20% (8/41) of papers recorded the duration poststroke of the patients at the time of the study [22,37-41,49,50]. The combined standard deviation (25.2 months) was greater than the combined mean (12.8 months), indicating the wide range of duration poststroke.

#### Outcome Measures

Outcome measures are documented and categorized to give an overview of how clinical, technical, and home use aspects, if any, were assessed in the literature. The measures were categorized as either measures of patient activity, balance, motor function, and quality of life or measures of system usability or other technical aspects. Most papers used multiple measures under different categories. Activity outcome measures assessed the ability of patients to perform activities (ie, exercise tasks and activities of daily living). Balance measures assessed the balance ability of patients, motor function measures assessed physical function capabilities, and quality of life measures assessed the quality of physiological and psychological well-being of patients. System usability measures assessed the usability of Kinect-based systems. Other technical measures, variables, or methods assessed the accuracy, design, and reliability of the K-SRS used. Multimedia Appendix 6 shows the measures categorized (Tables E-J) and ranked according to studies that used them.

#### Person-Generated Health Data

This section focuses on the data generated by patients and other study participants (such as healthy volunteers) through their use of K-SRS. Study descriptions of the papers can be found in Multimedia Appendix 7.

##### What Data Did People Generate by Using a Kinect-Based Stroke Rehabilitation System?

The types of data generated by patients when using a K-SRS can be broadly categorized as human performance data or system variable data. These PGHD were, in most cases, not provided to patients as feedback. The types of feedback provided to patients are described in the next section. Human performance data (n=25) are those used to indicate movement or exercise performance of the individual [22,36-40,42,45-48,52, 53,57,59,60,62,64-67,69-72]. Most of these data were generated directly from the Kinect sensor; others were from different sensors such as accelerometers. System variable data (n=20) were unsynthesized to indicate system or patient performance [23,30,35,39,41,44,49,50,53-56,58,60,61,63,67-69,71]. These types of data were used to evaluate a system's accuracy, feasibility, reliability, and effectiveness. Many papers generated such data directly from Kinect-based systems (n=12) [23,30,39,41,44,49,55,56,61,63,68,69]. Ten papers generated data from other sensors [35,50,53,56,58,60,61,67,69,71] such as an inertial wrist strap, 6 papers reported both performance and variable data [39,53,60,67,69,71], and 2 papers did not report data from individuals’ use of the rehabilitation systems [43,51]. For more detailed descriptions of these data, please see Multimedia Appendix 7 (Table K).

##### How Did People Have Access to Their Data?

**Figure 3:**
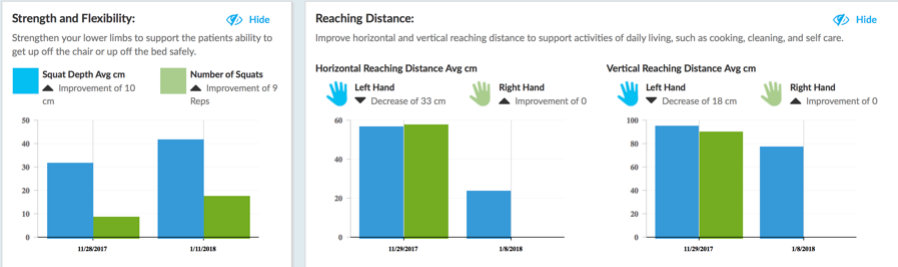
Clinician view: patient-generated health data outcomes summary.

**Figure 4:**
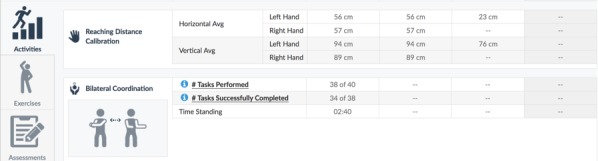
Clinician view: detailed patient performance data.

**Figure 5:**
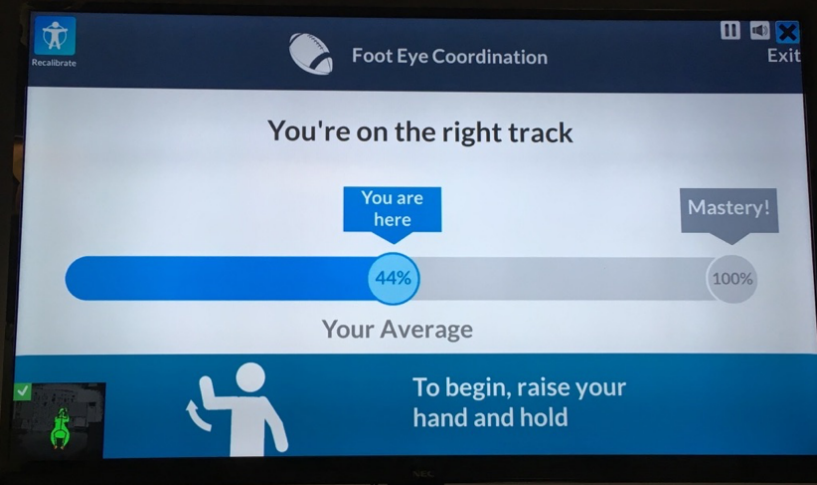
Patient view: sample postexercise game score.

People were provided with various forms of feedback, but in no cases did they access their complete data, data similar to those seen by their attending clinicians, from the K-SRS. For example, while clinicians may see a patient’s calculated reaching distance, patients would only see game task scores. It is unknown why this is so; of the 33 papers that provided feedback, only 11 papers provided reasons for giving any feedback at all. These included following good game design for a better user experience [67,70], guiding movements [49,56,60,61,69,70], reducing user errors [35,60,63], and assisting users in meeting exercise goals [35,46,50]. Figures 3 and 4 show sample clinician views, while Figure 5 shows a sample patient view after an exercise illustrating one of the systems in use [71,72].

The types of feedback provided can be categorized as guidance, progress, or task scores. Guidance feedback (n=19) is in the form of visual and auditory information, intended to facilitate performing an exercise or task. In 13 papers, patients were guided in performing a task through a visual interface [44,45,49,52,53,60-62,64,67,70-72]; in 1 paper, through auditory feedback [30]; and in 5 papers, through both visual and auditory guidance [23,35,54,56,66]. Progress feedback (n=4) tracked patient progress in terms of number of exercises or tasks completed or to be completed [42,46,63,65]. Task score feedback (n=10) was in the form of game scores provided as-is, without any interpretation of the user’s performance. These papers simply provided people with their scores at the end of a task execution [22,37-39,47,50,55,57,59,69]; 8 papers did not describe provision of feedback or any other mode of patient access to their data [36,40,41,43,48,51,58,68]. For more detailed descriptions of these data, please see Appendix 7 (Table L).

##### Who Else Used the Data and for What Purposes?

Use of PGHD can be categorized based on the purpose of use, which was for patient benefit, comparison of effects, assessment of K-SRS, or evaluation of other technologies. For patient-benefit use papers, 63% (12/19) were in the form of therapists using the data to prescribe or tailor rehabilitation to individual patient needs [23,46-49,58,65-67,69,70,72]. Comparison papers used PGHD for researchers to study the different effects of a K-SRS in different groups of people [57,72]. Use of K-SRS assessment research (n=13) was done to study system effectiveness, feasibility, accuracy, or reliability [22,23,30,41,42,44,45,50,54-56,61,71]. PGHD use for evaluation of other technologies employed the generated data to assess other technologies used in their K-SRS [35,40]; 5 papers used data for 2 purposes [23,35,56,61,72] and 10 papers did not describe use of PGHD [36-38,43,51,53,59,60,62,63]. For more detailed descriptions of PGHD use, please see Appendix 7 (Table M).

##### What Effects Were Reported From People’s Use of Their Own Data?

Only 1 paper [22] described any effects on a patient from using PGHD. This paper observed that when the patient was provided with her performance scores daily she remembered them and was motivated to improve the next day.

## Discussion

### Principal Findings

No prior systematic reviews have examined the literature for evidence about use or management of the patient health data that K-SRS users generate. In our own review of the K-SRS literature, we found that while more than three-quarters of the papers used PGHD in some way, only 1 described the effects of PGHD use [22]. Moreover, the fact that use was mainly for technical evaluation and secondarily for clinicians to prescribe exercises shows that patient participation was not a priority in the design of K-SRS. Additional evidence of this can be found in the data access provided to patients, which was mainly in the form of feedback to provide guidance. The focus of data provision has been to prescribe tasks and guide patients to perform movements rather than to allow patients to access and make sense of their own performance data. This represents a missed opportunity from the literature to engage poststroke patients in their own health care, as it has been shown that when patients have direct access to their PGHD they become more engaged and improve their health outcomes [7,9,10,73]. The lack of patient access to data also suggests that patient-centered design was not part of developing these Kinect-based systems [74], a key consideration in a modern participatory health paradigm. This factor could overlook PGHD and undermine the rehabilitation experience of patients [75].

The use of data overwhelmingly for technological development and assessment is clearly shown by 78% (32/41) of papers having a technical primary objective. Even if we acknowledge that it is necessary to assess the accuracy and reliability of Kinect-based systems, this technical focus confirms the finding of Webster et al [26] that this field of rehabilitation is still in its infancy. This presents an opportunity and challenge to evaluate clinical outcomes [25-27] (eg, effectiveness and safety of such systems), a challenge that only a few papers have taken up [36-39,49,50,57,71,72].

The focus of existing K-SRS papers was on upper extremities. It is interesting that while Kinect has the ability to track the whole body, only upper extremity software is described [25]. None of the papers in this review used lower extremity outcome measures.

Results of this review show that there is insufficient attention given to PGHD from K-SRS. While most studies provide some form of feedback, they do not allow patients to actively engage with data about their own rehabilitation, nor do the papers try to understand the health behavior impact of providing data access to patients.

### Limitations

While most papers described the data types collected in their papers and available feedback, often they were glossed over in the descriptions and discussion. As mentioned previously, this shows a lack of attention to PGHD and to health data management generally in K-SRS papers. The lack of documentation may also have limited the details into PGHD this review gathered (ie, some other benefit of PGHD may have occurred but was not documented or described). As such, while this review attempted to provide a snapshot of the PGHD types, access, and benefits, it may be incomplete. In short the lack of attention given to PGHD in the papers confirms the need for papers to pay attention to the PGHD of their K-SRS but also limits the PGHD evidence obtained. Due to time constraints, the authors of the papers reviewed were not directly contacted for more information on the PGHD they have given patients.

With regard to patients recruited, the majority of patients had infarct or ischemic type of stroke, and patients were not separated based on their stroke type. Given that there is some evidence that hemorrhagic patients benefit from rehabilitative therapies faster than infarct and ischemic patients [76], this could produce some stroke-type bias in the effectiveness results, where results derived largely from infarct patients are generalized for hemorrhagic patients as well.

### Conclusions

Reviewing current K-SRS literature through the lens of PGHD showed that there is a significant gap in our understanding of what it may contribute to the experience of patients who use K-SRS. Most papers provide some feedback but do not allow patients to engage with all of their PGHD (eg, for self-management of their health journey). This provides further evidence of the need for studies that contribute to the theoretical foundation of PGHD use [1]. It is also indicative of the need for future researchers of technology-based rehabilitation to consider PGHD and patient access to information in their system design and implementation. Improving our understanding of the effects of using PGHD could help in designing systems where the benefits of PGHD access are made available to patients. This paper calls for future studies on K-SRS—and studies that have the potential for generating patient health data in general—to pay more attention to how those data may influence the process of care.

## Appendix

Multimedia Appendix 1 Search strategy for part 1: analysis of systematic reviews.

Multimedia Appendix 2 Search strategy for part 2: review of person-generated health data use in Kinect-based stroke rehabilitation systems.

Multimedia Appendix 3 Objectives, methods, and structure of existing systematic reviews.

Multimedia Appendix 4 Analysis of person-generated health data, home use, and effectiveness in existing systematic reviews.

Multimedia Appendix 5 Types of papers.

Multimedia Appendix 6 Outcome measures.

Multimedia Appendix 7 Review of person-generated health data.

## Abbreviations

K-SRS: Kinect-based stroke rehabilitation systems
PGHD: person-generated health data
PRISMA: Preferred Reporting Items for Systematic Reviews and Meta-Analyses
