# Supplementary material for: Person-Generated Health Data in Simulated Rehabilitation Using Kinect for Stroke: Literature Review
Source: JMIR Rehabil Assist Technol. 2018 May 8;5(1):e11. doi: 10.2196/rehab.9123 (PMC5964303; doi:10.2196/rehab.9123)
Supplement: Multimedia Appendix 1 [file rehab_v5i1e11_app1.pdf]

Search strategy for Part 1, analysis of systematic reviews.

This is a Multimedia Appendix to a full manuscript published in the J Med Internet Res. For full copyright and citation information see http://dx.doi.org/10.2196/jmir.9123.

Search Strategy

The search phrase used was: *kinect AND review*. Search was conducted through six databases: IEEE Xplore, and ACM Digital Library for sources from the computing and engineering field; PubMed, and Biomed Central for sources from the health field; and the Cochrane, and Campbell Collaboration digital libraries to search for detailed reviews. Finally, Google Scholar was also utilised for a broader search of revelant papers [77,78]. It was necessary that the search returned reviews that mentioned Kinect, thus *all text* searches were conducted. For Google Scholar, only *all title* search was conducted. There was no date restriction. The search was closed in June 2017. The quality of the papers were not assessed, as it was not deemed critical to understanding PGHD access and utilisation in studies that utilised K-SRS.

The search returned 192 papers in total. GLD reviewed the titles of the papers, and their abstracts when necessary. Non-systematic reviews, or reviews of Kinect for non-rehabilitation purposes, were excluded (183 papers). The remaining nine were reviewed by GLD in full using the inclusion and exclusion criteria in the next section, where six more papers were excluded; resulting in the three systematic reviews that were analysed in this paper. This search process is illustrated below (Figure 1).

Figure 1. Search process for Part 1.

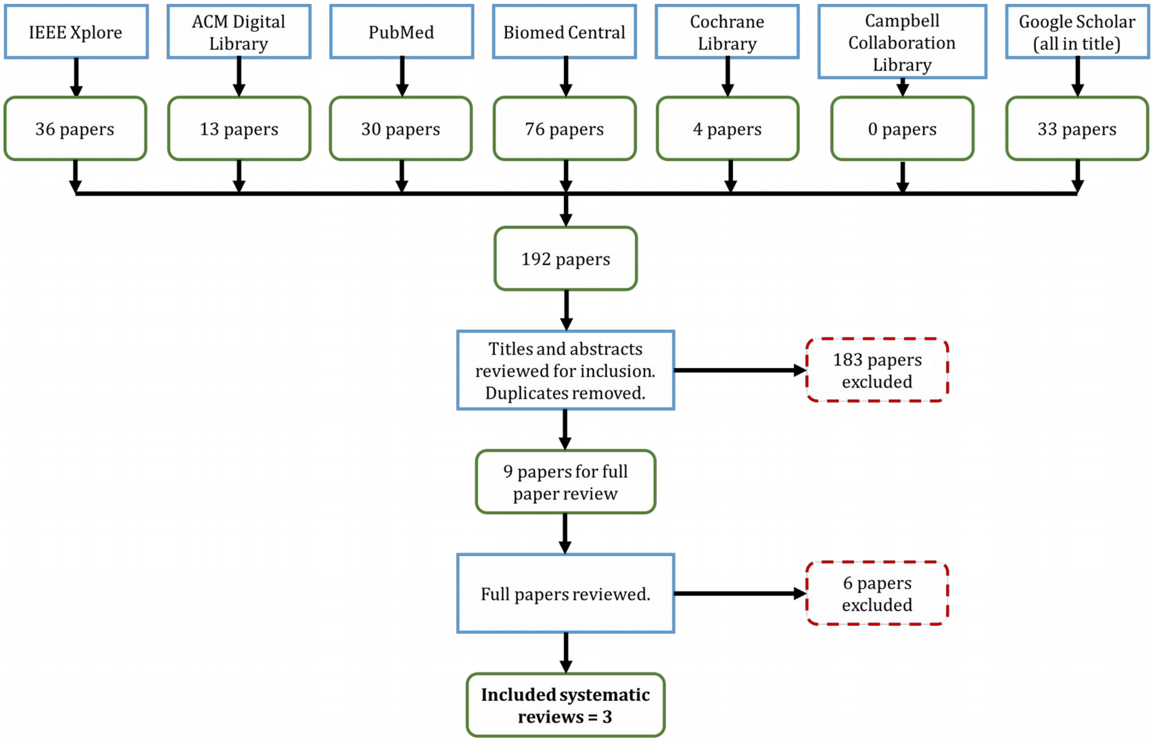

Inclusion and Exclusion Criteria

The inclusion and exclusion criteria (Table 1) ensured that the systematic reviews were focused on K-SRS, and examined articles in sufficient detail. This is necessary to meet the three points for analysis in part 1: PGHD utilisation, home based rehabilitation, and effectiveness. Papers were included if they reviewed systems primarily for stroke rehabilitation and used Kinect. Meanwhile, reviews for non-rehabilitation purposes such as measuring clinical outcomes, or assessing Kinect's gesture recognition capability were excluded. Broad scoping reviews that mainly took inventory of a general suite of technologies, and thus did not describe Kinect-based systems in detail, were also excluded.

Table 1. Inclusion and exclusion criteria for part 1.

Inclusion Criteria	Exclusion Criteria
Written in English.	Reviews for non-stroke rehabilitation purposes, e.g., assess Kinect's gesture recognition.
Systematic or literature reviews.	Broad scoping reviews that primarily took inventory of a suite of technology-based rehabilitation systems.
Reviews of systems that used Kinect.	
Reviews of systems for stroke rehabilitation.	

Data Extraction and Analysis

The content of each systematic review paper was analysed based on (1) their method for analysis vis-à-vis their objectives; (2) their focus on utilisation of patient-generated health data, including feedback given to users or patients; (3) the extent to which the systems included in their review are usable at home, including the challenges and recommendations of implementing at home; and (4) the effectiveness of the systems in their review based on patient outcomes as well as technological limitations that may affect those outcomes.

The objectives, methods and structure of each systematic review are detailed in Table 3 (Appendix 3). A summary of these systematic reviews vis-à-vis the themes of interest above can be found in Table 4 (Appendix 4).

---

77. Walters W. Google Scholar coverage of a multidisciplinary field. Information Processing and Management. 2007;43(4):1121-32. DOI: 10.1016/j.ipm.2006.08.006
78. Falagas M, Pitsouni E, Malietzis G, Pappas G. Comparison of PubMed, Scopus, Web of Science, and Google Scholar: strengths and weaknesses. The FASEB Journal. 2007;22(2):338-42. DOI: 10.1096/fj.07-9492LSF
