# Supplementary material for: Person-Generated Health Data in Simulated Rehabilitation Using Kinect for Stroke: Literature Review
Source: JMIR Rehabil Assist Technol. 2018 May 8;5(1):e11. doi: 10.2196/rehab.9123 (PMC5964303; doi:10.2196/rehab.9123)
Supplement: Multimedia Appendix 2 [file rehab_v5i1e11_app2.pdf]

Search strategy for Part 2, review of PGHD use in K-SRS.

This is a Multimedia Appendix to a full manuscript published in the J Med Internet Res. For full copyright and citation information see http://dx.doi.org/10.2196/jmir.9123.

Search Strategy

The search was conducted in four major databases: IEEE Xplore, ACM Digital Library, PubMed, and Biomed Central. The terms used were “Kinect”, “stroke”, “cerebrovascular accident”, “CVA”, and “rehabilitat*”. These were selected with the help of a University of Melbourne librarian. Keywords found in an initial search were also scanned for relevant terms (where “cerebrovascular accident” and “CVA” were discovered). The search phrase used was: *Kinect AND (stroke OR “cerebrovascular accident” OR cva) AND rehabilitat** for PubMed, Biomed Central, and IEEE Xplore. For ACM, the wildcard symbol “*” was not used as the resulting articles numbered more than 444,000, mostly unrelated. Rehabilitat* was instead replaced with the phrase “*rehabilitate OR rehabilitation*”. Reference lists on K-SRS of the reviews found in Part 1, were also included in the search for Part 2. There was no date restriction. The search was closed in June 2017. The quality of the papers were not assessed, as it was not deemed critical to understanding PGHD access and utilisation in studies that utilised K-SRS.

A total of 163 papers were found. GLD reviewed the titles of the papers, and their abstracts when necessary. 44 papers were excluded from this process. The remaining 119 papers were then examined by GLD in full using the inclusion and exclusion criteria outlined below. 78 papers were excluded in this phase, leaving 41 papers included for thematic analysis. This search process is outlined below (Figure 2).

Figure 2. Search process for Part 2.

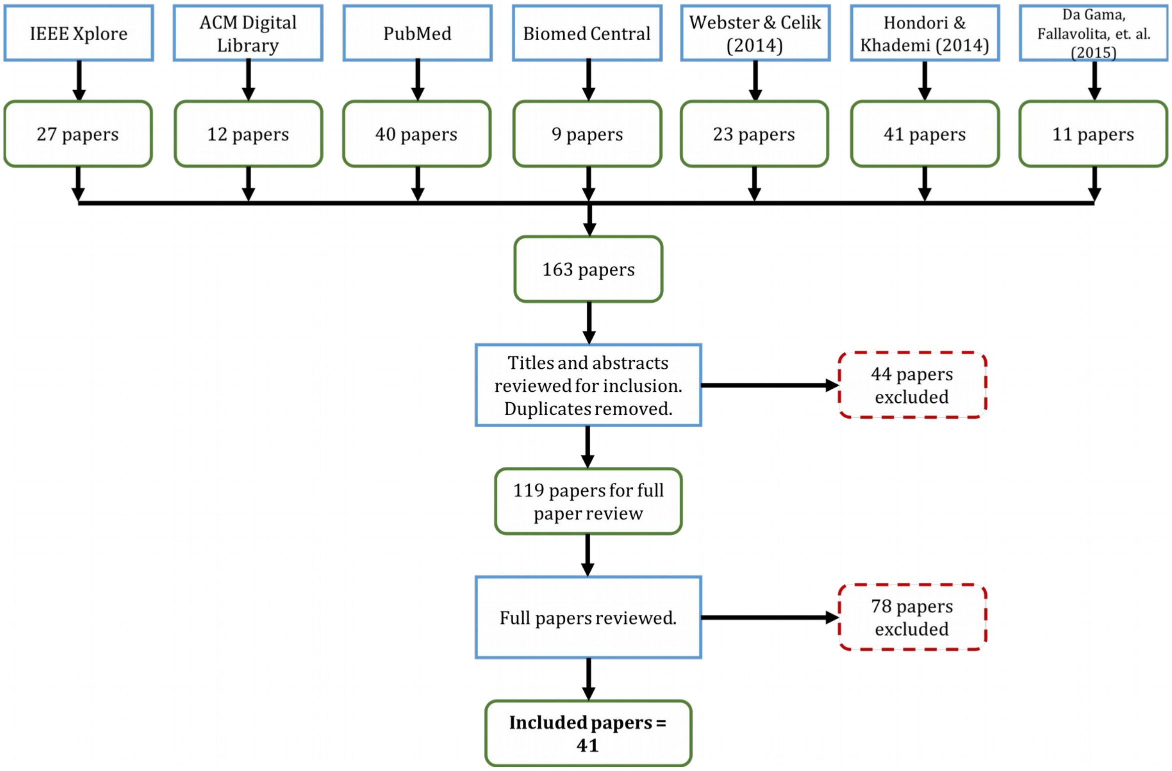

The quality of the papers were not checked prior to inclusion, as whether or not the papers were of high quality was not critical to understanding how PGHD were generated, accessed, utilised, and whether they resulted in any effects.

Inclusion and Exclusion Criteria

The inclusion and exclusion criteria for part 2 (Table 2) ensured that English full papers that presented rehabilitation systems for stroke, using Kinect, are included. Such systems may use other technologies as well, for example, in a rehabilitation environment that included the use of sensors. Papers were excluded if: their study focus was not on using or developing a rehabilitation system, e.g., evaluation of an algorithm; disease case was non-stroke; system did not use Kinect in any way; type was literature or systematic review; or if they were white papers. For papers that presented an update from previous publications, the latest paper was selected.

Table 2. Part 2 inclusion and exclusion criteria.

Inclusion Criteria	Exclusion Criteria
Full papers written in English.	Primary purpose of study is not on rehabilitation.
Presented rehabilitation systems for stroke.	Stroke was not the primary disease case.
Kinect was used for the system.	Kinect was not used in any way.
	Literature or systematic reviews.
	White papers.

Data Extraction and Analysis

Data were extracted from the papers based on a structured form that included the participants, outcome measures, and PGHD focus. For the focus on PGHD, the papers were analysed using the following questions: 1) What types of data did patients generate? 2) Did they have access to their PGHD, and if yes in what form? 3) How were these data utilised by patients, clinicians, developers and/ or researchers? 4) What effects were observed from PGHD utilisation? Appendix 6 shows the outcome measures categorised, and ranked according to references that used them. Detailed data extracted for the focus on PGHD can be found in Appendix 7.

To assist future studies in assessing the clinical effectiveness of K-SRS, papers were also categorised as either clinical- or technical-focused. The list of papers categorised according to their clinical/technical type is in Appendix 5 (Tables 5 and 6).
