# Supplementary material for: Person-Generated Health Data in Simulated Rehabilitation Using Kinect for Stroke: Literature Review
Source: JMIR Rehabil Assist Technol. 2018 May 8;5(1):e11. doi: 10.2196/rehab.9123 (PMC5964303; doi:10.2196/rehab.9123)
Supplement: Multimedia Appendix 3 [file rehab_v5i1e11_app3.pdf]

## Objectives, methods, and structure of existing systematic reviews of Kinect-based stroke rehabilitation systems.

This is a Multimedia Appendix to a full manuscript published in the J Med Internet Res. For full copyright and citation information see http://dx.doi.org/10.2196/jmir.9123.

Table 3. Summary of existing systematic reviews.

| Summary | Webster and Celik (2014) | Hondori and Khademi (2014) | Da Gama, Fallavollita, Teichrieb and Navab (2015) |
|---|---|---|---|
| Objectives | Gather relevant information on Kinect-based systems for stroke and elderly care. | Review notable motion capture systems. Term *notable* not specified. | Uncover research status of Kinect as a body-tracking tool for motor rehabilitation. Term *status* not specified. |
| Methods | | | |
| Databases | IEEE/IET Electronic Library, PubMed, ACM Digital Library, Computer Science Index, Safari Tech Books Online, and ISIS Web of Science. | PubMed, Google Scholar (title only). | IEEE Xplore, PubMed. |
| Inclusion | English, peer-reviewed papers directy or indirectly related to stroke or elderly care. This meant other reviews were included. | Not indicated. | English papers that are more than four pages in length, and which described a system for assistive interaction, clinical evaluation, or evaluation and improvement of Kinect's movement recognition and tracking. |
| Exclusion | No exclusion criteria. | Not indicated. | No exclusion criteria. |
| Search terms | Kinect (and combined sets of), stroke, rehabilitation, gesture, posture, clinical, geriatrics, elderly, ageing, aged, alert, fall, gait, exergame, serious game. | Kinect, rehabilitation | Kinect AND rehabilitation. |
| Analysis/ Structure | Categorised papers into stroke rehabilitation, with sub-categories spatial accuracy assessment, and methodological study; elderly care, with sub-categories falls detection or falls risk reduction; and serious/exercise games. | Four main points of discussion were rehabilitation systems using devices before the advent of Kinect; accuracy and stability considerations for Kinect developers; discussion on Kinect-based systems with or without patients; and other body-tracking sensors similar to Kinect. | Categorised papers into assistive, for papers that described systems for assistive interaction; evaluation, for papers that evaluated a measure, or used a measure; applicability, for papers that recruited patients; validation, for papers that used gold standard measures; and improvement, for papers that presented a technical improvement in design or implementation of a system. |

| | | | |
|---|---|---|---|
| Visual Presentation of Data | Tables summarised subject demographics and number (if available); descriptions of systems; measures used (if available); and main findings. For serious/exercise games, feedback provided was very briefly described, e.g., warnings, and game scores. | Tables and graphs. Table columns for papers that recruited patients were targeted disability, study type, purpose, evaluation, conclusion. Table columns for studies without patients were one-liner findings, comparison of depth sensors.<br>There were also four graphs to show number of papers in PubMed that mentioned Kinect alone, then Kinect and rehabilitation; and number of papers in Google Scholar that mentioned Kinect alone, then Kinect and rehabilitation. | Tables that summarised the tracking software used (Microsoft Software Development Kit/Open NI); movement analysed (e.g., any movement, upper limbs, hand trajectory or reach); system interface/visualition and feedback; features (e.g., therapist-configurable, auto-reporting of results to clinician); measure that was evaluated/used to evaluate; technique in improving the system; user evaluation (type of evaluation, e.g., survey, pre- and post-study); type (virtual/guidance/interactive game); target (stroke/other neurologic diseases); and results of the system (e.g., detection of correct exercises). |
| Results/ Discussion | Kinect has potential for physical and mental benefits, i.e., faster and better supported rehabilitation as well as enjoyability. Kinect-based systems can also extend guidance and correction of patient movements. Main limitations noted is on technological capability of Kinect, e.g., its difficulty to track fine motor movement such as fingers. | Kinect and other depth-based tracking sensors are better than earlier RGB-based sensors. Kinect's weakness lies in occlusion, in that sometimes other objects are mistaken as part of the user's limbs. However, with proper calibration Kinect was shown to be more precise and sensitive than other systems. It is particularly best for whole-body tracking. One study also showed that patients preferred Kinect over other off-the-shelf, consumer body-tracking devices Nintendo Wii and PlayStation 3 Move. | Interaction in the papers were mostly in the form of avatars, or game characters.<br>Popular movement exercises were reaching exercises, and mimicking movements in a screen.<br>For home interactive systems, there is a need to assess the capability of such systems to assess patient performance of exercises, and comparatively evaluate Kinect-guided movements vis-à-vis clinical measures.<br>Studies with patients reported that patients tended to perform exercises more often with Kinect-based systems because of the game/fun component.<br>Limitations of Kinect included occlusion; accuracy based on user position (standing), and movement (planar motions); and hands are depicted only as a point in space, hence finger |

| | | | |
|---|---|---|---|
| | | | movement tracking is impossible, unless Kinect is hung from above, focused on the hands alone, and trained to recognise the fingers. |
