# Supplementary material for: Person-Generated Health Data in Simulated Rehabilitation Using Kinect for Stroke: Literature Review
Source: JMIR Rehabil Assist Technol. 2018 May 8;5(1):e11. doi: 10.2196/rehab.9123 (PMC5964303; doi:10.2196/rehab.9123)
Supplement: Multimedia Appendix 4 [file rehab_v5i1e11_app4.pdf]

Analysis of PGHD, home use, and effectiveness in existing systematic reviews of Kinect-based stroke rehabilitation systems.

This is a Multimedia Appendix to a full manuscript published in the J Med Internet Res. For full copyright and citation information see http://dx.doi.org/10.2196/jmir.9123.

Table 4. Existing systematic reviews vis-à-vis themes of interest.

Themes	Webster and Celik (2014)	Hondori and Khademi (2014)	Da Gama, Fallavollita, Teichrieb and Navab (2015)
Did the review analyse PGHD utilisation in the literature?	No	No	No
Did the review analyse home use in the literature?	No	No	No
Did the review analyse effectiveness in the literature?	Yes	Yes	Yes
