# Supplementary material for: Person-Generated Health Data in Simulated Rehabilitation Using Kinect for Stroke: Literature Review
Source: JMIR Rehabil Assist Technol. 2018 May 8;5(1):e11. doi: 10.2196/rehab.9123 (PMC5964303; doi:10.2196/rehab.9123)
Supplement: Multimedia Appendix 5 [file rehab_v5i1e11_app5.pdf]

Types of papers.

This is a Multimedia Appendix to a full manuscript published in the J Med Internet Res.
For full copyright and citation information see http://dx.doi.org/10.2196/jmir.9123.

Table 5. Clinical-focused papers.

Clinical-focused papers (N=9)	
Reference	Description
Cohort Studies (N=2)	
[36]	Compared the physical and psychological effects on patients who either used a Kinect-based rehabilitation, or a bicycle ergometre training system.
[57]	Compared the effects of using Kinect-based training games on stroke patients with varied cerebral hemisphere-affected areas.
Case Reports (N=2)	
[49]	Evaluated a developed reaching exercise game for clinical effectiveness with 15 patients in a non-controlled setting.
[50]	Evaluated the clinical effectiveness of an off-the-shelf Kinect-based game for rehabilitation. Changes in the activated brain regions were also observed.
Randomised Controlled Trials (N=5)	
[71]	Study protocol: will evaluate the feasibility, efficacy and saftey of a Kinect-based system.
[72]	Study protocol: will assess the impact on patients who will use an interactive system at home, with a traditional home exercise programme.
[37,38]	Compared the effects of using Kinect-based games along with usual therapy on patient muscle strength and tone, against patients who only performed traditional therapy.
[39]	Assessed the clinical effectiveness of a tele-motion rehabilitation system by comparing the effects on patients who used the system, against those who performed traditional home-based exercises.

Table 6. Technical papers.

Technical papers (N=32)	
Reference	Description
Survey (N=1)	
[51]	Used a questionnaire to gather user perceptions, and preferences to design a mode of interaction with a visual object in an augmented reality environment.
Proof of concept (N=5)<br>*Tested the concept of K-SRS, and using other technologies with Kinect, for stroke rehabilitation.*	
[48]	Assessed the feasibility of using a gamified, home-based rehabilitation system for stroke patients.
[40]	Investigated the feasibility of using functional electrical stimulation to upper limb muscles for completion of tasks; and using iterative learning control algorithms to control the FES signals applied to the muscles.
[45]	Tested the concept of using body machine interfaces with Kinect.
[41]	Tested a Kinect-based model for assessing individual components of the timed-up and go test, followed by an assessment of the system's reliability.
[61]	Assessed the feasibility of a system for robot-assisted rehabilitation through gaze tracking, a brain-computer interface, and Kinect.
Application Development (N=17)<br>*Primarily described the process of designing or building a system for stroke rehabilitation.*	
[22,35,43,47,52,56,63,64,65,	Developed a system to provide movement or posture therapy.<br>*Additionally*: [66] included a monitoring component for falls prevention among the elderly;

68]	[47] provided speech therapy; [43] re-engineered a previously-developed immersive multimedia rehabilitation environment for improved control; and [56] used ‘intelligent objects’ with sensors to aid in ADL recovery.
[46,55]	Developed a system to assess movement capabilities of patients.<br>*Additionally:* [46] provided rehabilitation for motor function.
[23]	Developed a system to assist patients in improving movement, and predicted their recovery.
[53]	Developed a hybrid system that incorporated a low cost brain computer interface system called MindWave for brain signal input, with Kinect tracking user movement.
[65]	Digitised box-and-blocks test, where a traditional BBT was used but the progress, quantified by the number of blocks moved within a period of time, was tracked by Kinect.
[42]	Virtualised BBT, with patients controlling a virtual hand to move virtual blocks.
[69]	Proposed an exercise game system framework that provides online biofeedback to patients and therapists based on patient movements for safer and better exercise sessions.
Platform Development (N=6)<br>*Explained how multiple body-tracking technologies could be integrated in one system.*	
[54,70]	Developed platforms that made use of multiple consumer body-tracking devices, e.g., Kinect and Nintendo Wii, as input devices.
[59]	Developed a platform that made use of multiple consumer body-tracking devices, i.e., Kinect, Leap Motion, and Orbotix Sphero to be used simultaneously to track multiple parts of the body.
[67]	Offered gamified rehabilitation exercises to patients (which therapists can prescribe], monitor their performance, and connect with online health records to share information with clinicians; while wearables send updates for regular monitoring of patients' well-being.
[58]	Developed a K-SRS, and a cuffless blood pressure device with a chair-based electrocardiogram and photoplethysmograph for monitoring.
[60]	Developed a smart glove in-house to work with Kinect and track fine motor finger movement.
Assessment (N=3)<br>*Conducted technical assessments of Kinect as a body-tracking device for stroke rehabilitation.*	
[44]	Assessed its tracking and evaluation of components of spatiotemporal gait movements.
[30]	Assessed Kinect’s test-retest reliability in finding movement indices.
[62]	Compared the precision of Kinect against another device, Shimmer, in tracking exercise movements.
