# Supplementary material for: Person-Generated Health Data in Simulated Rehabilitation Using Kinect for Stroke: Literature Review
Source: JMIR Rehabil Assist Technol. 2018 May 8;5(1):e11. doi: 10.2196/rehab.9123 (PMC5964303; doi:10.2196/rehab.9123)
Supplement: Multimedia Appendix 6 [file rehab_v5i1e11_app6.pdf]

Outcome measures.

This is a Multimedia Appendix to a full manuscript published in the J Med Internet Res. For full copyright and citation information see http://dx.doi.org/10.2196/jmir.9123.

The outcome measures presented are categorised, and ranked according to the number of papers that used them as measures.

Table 7. Technical measures.

Technical Measures (Reference count =18)	
Outcome Measures	Reference
To assess accuracy (Ref. count =13)	
Comparison of cursor movement with Trunk Impairment Scale results	[45]
Frequency stimulation and refresh rate	[54]
Kinect-tracked Timed-Up and Go Test variables	[41]
Number of movements tracked by Kinect versus Shimmer	[62]
Standing, sitting, and lying posture variables	[64]
Dynamic Time Warping	[23,55]
Sensor ability to track posture	[66]
System validation tests	[67]
Analysis of personal dead reckoning algorithm in tracking body and hand/fingers	[60]
Arbitrary direct observation of system errors	[58]
Analysis of sensor data attached to 'intelligent' objects	[56]
Images over time to track occlusion and gaze tracking of moving objects	[61]
Computational speed of tracking algorithm	[61]
Comparison of gaze, proxy, and end-effector positions	[61]
To assess system design (Ref. count =4)	
Feedback questionnaire	[22]
User requirements	[52]
Semi-structured interviews	[40]
User workflow	[67]
To assess reliability (Ref. count =3)	
Standard Error of Measurement and Minimum Detectable Change	[41]
Kinect-tracked kinematics variables	[30]
Event-related desynchronisation and scalp activation maps	[61]
Classifier performance of patients	[61]
Support vector machine classifier	[61]
Velocity of the brain-controlled interface signals	[61]
To predict patients' recovery progress (Ref. count =1)	
Auto-Regressive Integrated Moving Average model	[23]
Observe rehabilitation effects on brain (Ref. count =1)	
Brain Regions of Interest activated	[50]

Table 8. Motor function measures.

Motor Function Measures (Reference count = 14)	
Outcome Measures	Reference
To assess upper extremity movement (Ref. count =13)	
Fugl-Meyer Upper Extremity Assessment	[22,38-40,42,47,48,50,72]
Box-and-Blocks Test	[38,42,65,72]

Action Research Arm Test	[23,40]
Motor Activity Log	[39,72]
Modified Motor Assessment Scale (Upper Arm)	[71]
Trunk Impairment Scale	[45]
Shoulder and Elbow Flexion data from the Qualisys Motion Capture System	[57]
Goniometre data	[38]
Kinect-tracked kinematics data	[23]
Brunnstrom Stage (haemiplegic arm and hand)	[42]
Korean National Institute of Health Stroke Scale	[42]
Chedoke Arm and Hand Activity Inventory	[39]
Wolf Motor Function Test	[50]
To assess muscle tone (Ref. count =2)	
Modified Ashworth Scale	[37,57]
To assess muscle strength (Ref. count =1)	
Manual Muscle Test	[37]

Table 9. Activity measures.

Activity Measures (Reference count = 7)	
Outcome Measures	Reference
To assess gait (Ref. count=3)	
Timed Up and Go Test	[36,41,44]
10-Metre Walking Test	
Step Test	[41,44]
To assess abilities of patients to perform activities of daily living (Ref. count =3)	
Canadian Occupational Performance Measure	[48]
Record of errors and error patterns	[35]
System-generated movement variables	[67]
To assess physical activity (Ref. count =2)	
Functional Independence Measure	[37,57]
To assess safety (Ref. count =1)	
Borg Rating of Perceived Exertion Scale	[71]
Visual Analogue Scale (pain & fatigue)	
To measure energy expenditure (Ref. count =1)	
ActivPal accelerometre	[71]

Table 10. System usability measures.

System Usability Measures (Reference count = 8)	
Outcome Measures	Reference
To assess user experience (Ref. count =4)	
IBM Game Experience Questionnaire	[48]
IBM System Usability Questionnaire	
Other questionnaires (not described)	[45,51,68]
To assess system usability (Ref. count =3)	
Reasons of patients for missing sessions	[71]
Use eye gaze and duration qualitative data, utterances or verbal responses, general body actions, and initiated actions	[43]
User feedbacks (Method undescribed)	[46]
To assess feasibility (Ref. count =1)	

Ratio of session completion	[71]
To assess enjoyment and benefits (Ref. count =1)	
5-point likert scales	[71]
For economic evaluation (Ref. count =1)	
Incremental cost-effectiveness ratio	[72]
To assess system attractiveness and appeal (Ref. count =1)	
Semi-structured interviews	[48]

Table 11. Balance measures.

Balance Measures (Reference count = 6)	
Outcome Measures	Reference
To assess standing balance (Ref. count =4)	
Functional Reach Test	[41,71]
Lateral Reach Test (medio-lateral balance)	[71]
Standing Double-Legged Balance (eyes closed)	[44]
Standing Weight Bearing Asymmetry	
Berg Balance Scale	[49]
Tinetti Performance Oriented Mobility Assessment - balance	
Brunnel Balance Assessment	
To assess sitting balance (Ref. count =2)	
Standardised Four-point Sitting Balance Test	[71]
Medial-Lateral Weight Shifting Test	[44]
To assess mobile balance (Ref. count =2)	
Activities-Specific Balance Confidence Scale	[48]
Biofeedback Analysis from AP1153 BioRescue system	[36]

Table 12. Quality of life measures.

Quality of Life Measures (Reference count = 4)	
Outcome Measures	Reference
To assess psychological well-being (Ref. count =3)	
Beck Depression Inventory	[36]
Relationship Change Scale	
Korean Mini-Mental Status Examination	[42]
Patients self-log exercise time, feelings (qualitative), other health services received, and any adverse events	[72]
To assess quality of life (Ref. count =2)	
Stroke-Specific Quality of Life Scale	[48]
Stroke Impact Scale	[72]
