# Supplementary material for: Person-Generated Health Data in Simulated Rehabilitation Using Kinect for Stroke: Literature Review
Source: JMIR Rehabil Assist Technol. 2018 May 8;5(1):e11. doi: 10.2196/rehab.9123 (PMC5964303; doi:10.2196/rehab.9123)
Supplement: Multimedia Appendix 7 [file rehab_v5i1e11_app7.pdf]

Review of Person-Generated Health Data.

This is a Multimedia Appendix to a full manuscript published in the J Med Internet Res.
For full copyright and citation information see http://dx.doi.org/10.2196/jmir.9123.

Table 13. Data generated from systems.

What data are generated from using the system?	
Reference	Description
Performance Data (N=25)	
[22,36-39,46-48,53,57,59,60,69,70]	Generated data in the form of game scores, i.e., how much of a task was completed well by the user.
[52]	Recorded the number of incorrect positions executed by the user.
[62,64,67]	Recorded both correct and incorrect movement executions.<br>*Additionally*: [64] also recorded exercise history and progress; [67] also recorded patient compensation movements.
[66]	Generated task counts that recorded progress, posture duration, and errors committed.
[42,65]	Counted the number of blocks moved from one side to the other.<br>*Additionally*: [65] also recorded the hand movement accuracy and speed.
[40]	Used functional electrical stimulation in a platform that produced arm and shoulder reach and flexion data; and time to complete tasks.
[57]	Measured the shoulder and elbow flexion range from the Qualisys Motion Capture System.
Variable Data (N=20)	
[23,30,49,56,61]	Collected kinematic variables, which are data used for motion tracking. These data included hand velocity, acceleration, and jerkiness.<br>*Additionally*: [23] kept video recordings of users, movement distance and predicted stroke recovery duration; [56] recorded hand orientation, grasp, and haptic interaction; and [61] recorded gaze direction and objects selected, and motor imagery based on EEG signals.
[41]	Generated timed-up and go test variables, i.e., standing peak trunk flexion angle, standing angular velocity, lengths of first stride and first step, gait speed, turn time, and total TUG time, including the variables' standard error of measurement (SEM), and minimum detectable change (MDC).
[44]	Recorded 3D skeletal position data (ankles and shoulder centre), gait velocity, centre of pressure path velocity, force distribution (lower limbs).
[55]	Collected range of movement, position, and rotation values of users.

[39]	Collected shoulder flexion and abduction, elbow flexion, and compensatory movements.
[63]	Recorded the number of user attempts and an error summary.
[53]	Monitored brain signals from a brain computer interface and electroencephalographic data.
[58]	Monitored electrocardiogram and photoplethysmograph data as well as blood pressure.
[67]	Used wearble sensors to monitor inertial and acceleration data.
[60]	Used a smart glove they developed to produce a detailed hand skeleton image that includes finger movement.
[71]	Study protocol: will use a wearable sensor to monitor energy expenditure.
[35]	Used weight and accelerometre sensors at the bottom of tea saucers and jugs to track errors and error patterns.
[54]	Used a training platform that consisted of the Nintendo Wii Balance Board, Microsoft Kinect, TEREFES electrosimulator, and a computer. Data generated from the platform included frequency stimulation and muscle synergies.
[68]	Color image, 3D depth image, and voice signal
[69]	Biosignals for muscle activity
[50]	Cortical activation pattern, blood-oxygen-level dependent signal volume

Table 14. Patient access to PGHD.

Did patients have acccess to their PGHD?	
Reference	Description
Guidance (N=19)	
[44,45,49,52,53,60-62,64,67,70-72]	Guided patients in performing a task through a visual interface.<br>*Additionally*: [67,70-72] also provided game scores, i.e., number/percent of tasks performed correctly; and [67] provided the compensatory movements patients made.
[30]	Provided auditory feedbacks.
[23,35,54,56,66]	Provided both visual and auditory guidance.<br>*Additionally*: [23] also provided game scores; [66] showed patients the duration they were able to hold a certain position; [54] provided patients their centre of pressure information as they stand on a balance platform; and [56] also provided tactile feedback.
Progress (N=4)	
[42,65]	Technologised the box-and-blocks test. Progress was tracked through the number of blocks patients needed to move from one side to the other.
[63]	Showed red and green balloons to show progression for uncompleted and completed tasks, respectively. Also showed

	users the number of attempts they took to do an exercise, as well as their errors.
[46]	Informed patients when they have met exercise goals.
Task Scores (N=10)	
[22,37-39,47,50,55,57,59,69]	All the papers simply provided scores at the end of a task execution.<br>*Additionally*: [39] showed patients the compensatory movements they made; [47] informed patients whether the images they selected were correct or not.

Table 15. PGHD utilisation.

How was PGHD Utilised?	
Reference	Description
Patient (N=19)	
[46,48,70]	Using data for therapists to prescribe or tailor-fit rehabilitation to individual patient needs through *calibrating game intensity or duration*.
[23,49,65,66,69]	Using data for therapists to prescribe or tailor-fit rehabilitation to individual patient needs through *prescribing appropriate exercises*.
[47,58,67,72]	Using data for therapists to prescribe or tailor-fit rehabilitation to individual patient needs through *monitoring and evaluation of patient progress*.
[35,39,49,52,56,61, 64,68]	Utilised PGHD primarily to guide patients as they perform rehabilitation exercises.<br>*Additionally*: [39] utilised data for evaluation of patient progress.
Comparison (N=2)	
[57]	Utilised the data to compare performance between four different groups of participants (20 patients - 10 left, 10 right hemiparesis; 20 healthy - 10 left, 10 right trained) on a set of exercises.
[72]	Will compare system-generated data with clinical outcome measures, e.g., Fugl-Meyer scores and BBT.
Kinect-based Systems (N=13)	
[22,55]	Utilised data to assess their Kinect-based systems on their effectiveness *of assessing patient improvement*.
[42,45]	Utilised data to assess their Kinect-based systems on their effectiveness *through evaluation of their system's performance as compared with traditional rehabilitation*.
[50]	Utilised data to assess their Kinect-based systems on their effectiveness *to activate significant brain regions*.
[23]	Assessed the feasibility of their system to predict the duration of patients to recovery.
[71]	Assessed both feasibility and effectiveness of their system.
[30,41,44,56]	Utilised PGHD to assess the reliability of their systems in tracking movement, and motor function of patients. [56] assessed reliability of interaction between their exercise games and intelligent objects.
[54,61]	Assessed their platform's accuracy, i.e., timing and synchronisation.
Other Technologies (N=2)	
[40]	Used PGHD to observe the effects of applying functional electrical stimulation to patient muscles as they attempt to complete functional tasks.
[35]	Used PGHD produced by weight and accelerometre sensors to record the errors patients make, and observe their error patterns.
